# Topiramate for the treatment of neonatal seizures and beyond

**DOI:** 10.1111/epi.18563

**Published:** 2025-07-31

**Authors:** Wolfgang Löscher, Janet S. Soul

**Affiliations:** ^1^ Translational Neuropharmacology Lab, NIFE, Department of Experimental Otology of the ENT Clinics Hannover Medical School Hannover Germany; ^2^ PrevEp, Inc. Bethesda Maryland USA; ^3^ Fetal‐Neonatal Neurology Program, Department of Neurology Boston Children's Hospital Boston Massachusetts USA; ^4^ Harvard Medical School Boston Massachusetts USA

**Keywords:** cognition, epilepsy, hypoxic–ischemic encephalopathy, perinatal asphyxia, phenobarbital

## Abstract

Acute symptomatic neonatal seizures are one of the most common neurological disorders in newborns admitted to neonatal intensive care units and require prompt treatment. Up to 50% of neonatal seizures are refractory to first‐line medications such as phenobarbital (PB), and another 30% fail second‐line therapy. Furthermore, antiseizure medications (ASMs) such as PB have short‐term adverse effects and may exert long‐term detrimental effects on neurodevelopment. Thus, the development of more effective and safer ASMs is an urgent medical need. Because of its multimodal mechanisms of action and neuroprotective activity as well as promising preclinical and clinical findings, topiramate (TPM) is currently among the most attractive ASMs for the treatment of PB‐refractory neonatal seizures. However, parenteral TPM is not clinically available, which restricts its use in most newborns with acute seizures. In this review, we critically discuss the current knowledge about TPM as a treatment for neonatal seizures and associated conditions. We describe both preclinical and clinical data and highlight that the neuroprotective activity of this drug, not shared by most other ASMs, may enhance the efficacy of therapeutic hypothermia to decrease adverse neurodevelopment after neonatal brain injury. In addition, we describe two novel intravenous formulations of TPM currently being developed for clinical use. One formulation uses the highly tolerable U.S. Food and Drug Administration (FDA)–approved excipient meglumine for the preparation of an aqueous TPM solution, so is particularly suitable for neonates. We recommend prospective randomized controlled clinical trials designed to test the safety and efficacy of intravenous TPM for neonatal seizures. TPM doses in such trials should be based on the maintenance of effective plasma levels not achieved in most previous clinical studies with enteral administration of TPM suspensions. Furthermore, the potentially beneficial neuroprotective effects of TPM on adverse outcomes associated with neonatal seizures and their etiologies should be examined in such trials.

**Key points**

- Topiramate is an attractive antiseizure medication (ASM) to test in neonatal seizures because of multiple mechanisms of action, neuroprotective effect, and proven efficacy in children.
- Preclinical data demonstrate neuroprotective effect by neuropathology and behavior in multiple animal models of hypoxia–ischemia, without excess apoptosis.
- Clinical studies in human newborns show promising effect of topiramate on refractory seizures and neonatal hypoxic–ischemic encephalopathy, without serious adverse effects.
- Pharmacokinetic data suggest that intravenous TPM should be tested at higher doses than used in most published clinical studies.
- Clinical trials of a novel intravenous TPM formulation should test the safety and efficacy of TPM for neonatal seizures, epilepsy prevention, and neuroprotection.

## 1 INTRODUCTION

**FIGURE 1:**
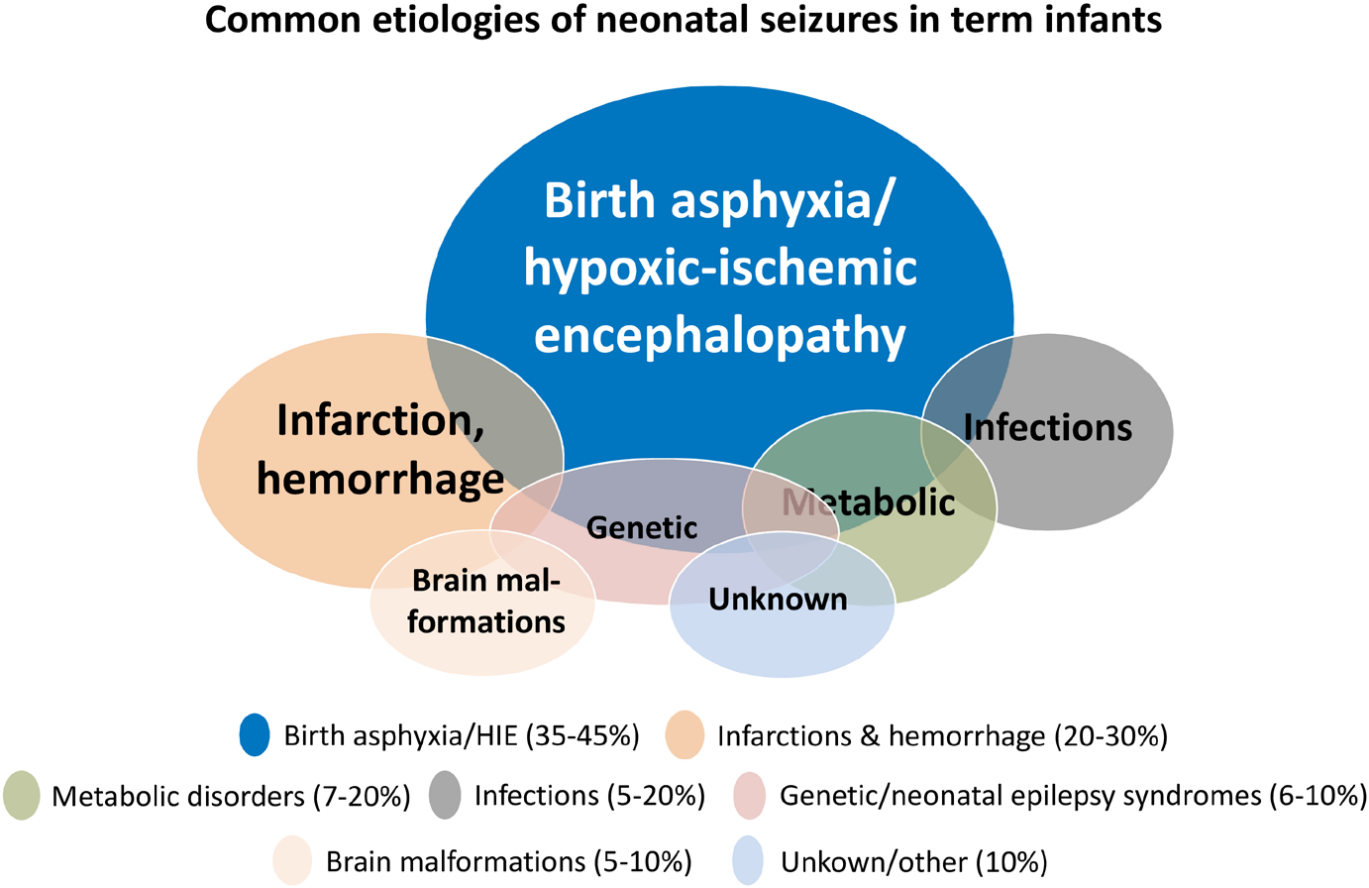
Relative occurrences of common etiologies of neonatal seizures in term infants. Modified from Pressler et al.^4^

Neonatal seizures are a common neurological emergency in newborn babies, arising in around 2–3 per 1000 term live births, and are more common in preterm infants.^1^, ^2^, ^3^ The majority of neonatal seizures are acute symptomatic and are caused by perinatal brain injuries, for example, hypoxic–ischemic encephalopathy (HIE) and perinatal arterial ischemic stroke.^2^ HIE usually results from perinatal asphyxia (PA) by the interruption of placental blood flow, the most common cause of neonatal seizures (Figure 1). HIE occurs in ~1.5 cases per 1000 live births and is one of the leading causes of neonatal death and adverse long‐term neurological outcomes.^5^

Neonatal seizures occur typically in the first 12–24 h after birth and require admission to the neonatal intensive care unit (NICU). The mortality associated with neonatal seizures ranges from 3.3% to 39.4%, with an overall mortality rate of 4%, necessitating rapid treatment.^6^ If not treated, neonatal seizures typically resolve within hours to days; however, these seizures exacerbate brain damage and have a negative impact on the prognosis of the patients, so rapid interruption of the seizures is essential.^7^ In term neonates the HIE is treated by therapeutic hypothermia (TH), which is standard of care in the NICU for this condition to reduce mortality and neurologic morbidity, while neonatal seizures are treated by antiseizure medications (ASMs), which are administered during TH in term neonates or without TH in pre‐term infants.^3^, ^8^

**FIGURE 2:**
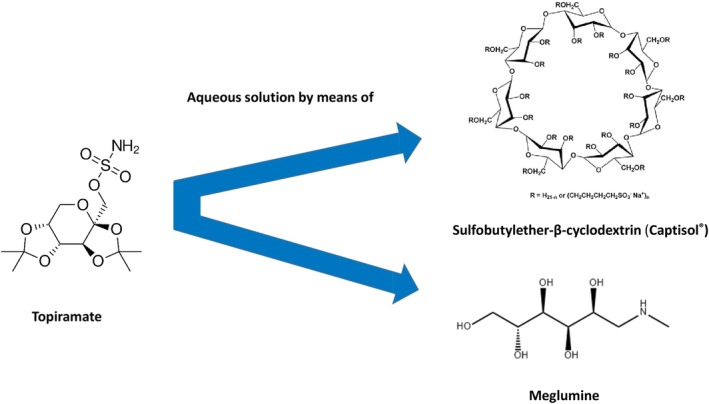
Structures of topiramate and the excipients sulfobutylether‐β‐cyclodextrin (Captisol) and the amino sugar meglumine (*N*‐methyl‐d‐glucamine). See text for details.

Phenobarbital sodium (PB; Sezaby) is the only ASM that is approved by the U.S. Food and Drug Administration (FDA) for this condition and is considered as first‐line treatment for neonatal seizures.^1^, ^9^ However, seizures are suppressed by intravenous (i.v.) administration of PB in only about 50% of cases, necessitating off‐label i.v. administration of second‐ and third‐line ASMs, such as fosphenytoin, midazolam, or levetiracetam (LEV).^10^, ^11^ As discussed in more detail below, topiramate (TPM), which is currently not available as i.v. formulation, has been administered by the nasogastric route in neonates after failure of first‐ and second‐line treatments, with promising results (Section 3.1). An added advantage is the neuroprotective effect of TPM, which may reduce the adverse outcome of PA/HIE (Section 2.3). Thus, pediatric neurologists considered TPM to be among the most promising ASMs for the treatment of PB‐refractory neonatal seizures, provided that an i.v. formulation becomes available.^10^, ^12^ Currently, two i.v. formulations of TPM are in the development pipeline (Section 4). One of these formulations, which uses a beta‐cyclodextrin (Captisol; Figure 2) as an excipient, has undergone initial pharmacokinetic and safety studies in adults.^13^, ^14^ The other formulation, which uses the amino sugar meglumine as an excipient (Figure 2),^15^ is currently developed for the treatment of neonatal seizures and received orphan drug designation (ODD) for this indication in both the United States and Europe.

In this review, we critically discuss the current knowledge about TPM as a treatment for neonatal seizures. We describe both preclinical and clinical data and highlight that the neuroprotective activity of this drug, which is not shared by most other ASMs, may enhance the efficacy of TH to decrease mortality and adverse neurodevelopment after HIE.

## 2 PRECLINICAL DATA

TPM, a sulfamate derivative of the naturally occurring sugar D‐fructose (Figure 2), was initially developed as an antidiabetic agent.^16^, ^17^ TPM was devoid of hypoglycemic activity, but the structural resemblance of its sulfamate moiety to the sulfonamide moiety in acetazolamide (and other arenesulfonamide anticonvulsants) prompted an evaluation of possible anticonvulsant effects.^16^ TPM proved to be effective in a wide array of rodent models of seizures and epilepsy, including models for focal‐onset and generalized seizures. Furthermore, TPM exerted neuroprotective activity in models of status epilepticus (SE) and ischemia, including hypoxia–ischemia (HI) in neonates.^18^ The broad spectrum of antiseizure and neuroprotective effects of TPM are a likely consequence of its multimodal mechanisms of action.

### 2.1 Mechanisms of action of TPM

**FIGURE 3:**
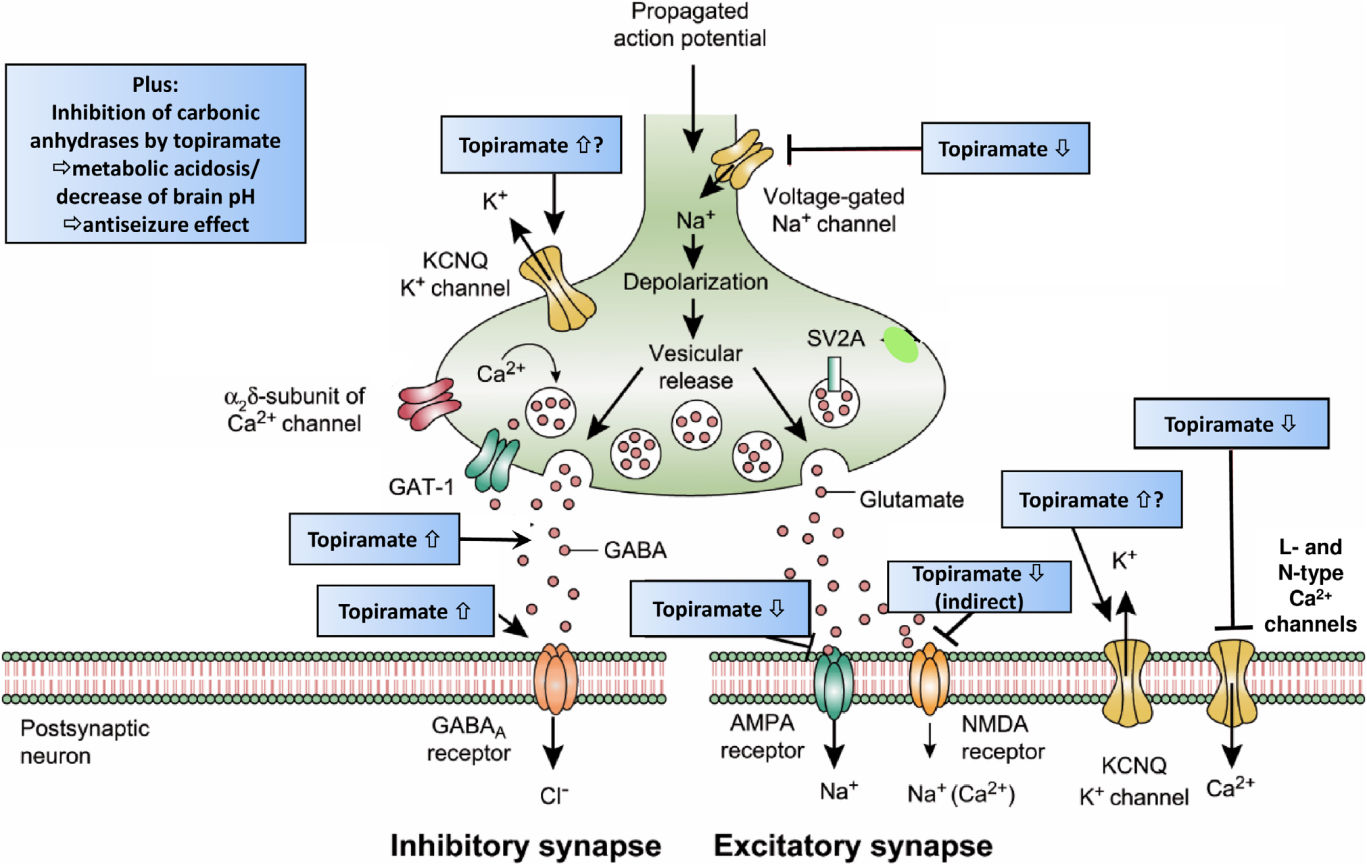
The multiple mechanisms of action of topiramate. The figure shows both an inhibitory (left‐hand side) and an excitatory nerve terminal (right‐hand side); note that, in reality, the same nerve terminal does not release both γ‐aminobutyric acid (GABA) and glutamate. AMPA, α‐amino‐3‐hydroxy‐5‐methyl‐4‐isoxazole propionic acid; GABA, γ‐aminobutyric acid; NMDA, *N*‐methyl‐d‐aspartate; SV2A, synaptic vesicle glycoprotein 2A. The figure was modified from Löscher and Klein.^19^ See text for details.

There are at least four established pharmacodynamic properties of TPM that are likely to contribute to its antiseizure and neuroprotective activities.^17^ These include inhibitory effects on voltage‐gated Na^+^ and Ca^2+^ channels, inhibitory effects on glutamate‐activated ion channels of the α‐amino‐3‐hydroxy‐5‐methyl‐4‐isoxazole propionic acid (AMPA) and kainate subtype, and positive modulatory effects on the γ‐aminobutyric acid (GABA)_A_ chloride ionophore complex (Figure 3). At least three more possible pharmacodynamic properties may also be involved, including a positive modulation of some types of voltage‐gated K^+^ channels, inhibition of some carbonic anhydrase isoenzymes, and modulation of the presynaptic neurotransmitter release process, resulting in reduced extracellular levels of glutamate (shown in spontaneous epileptic rats), and increased extracellular levels of GABA (shown in gerbils and humans).^17^ The effects of TPM on voltage‐gated Na^+^ channels include both the transient and the persistent Na^+^ current.^20^ Shank et al.^16^ postulated that the modulatory effects of TPM on most of these various protein complexes have a common mechanistic basis, that is, that TPM binds to certain membrane ion channel proteins at phosphorylation sites and thereby allosterically modulates channel conductance and secondarily inhibits protein phosphorylation. The combination of multimodal mechanisms of TPM illustrated in Figure 3 is unique and not shared by any other ASM.

### 2.2 Antiseizure effects of TPM in neonatal rodent models

Koh and Jensen^21^ were the first to report an antiseizure effect of TPM in a rodent model of neonatal seizures (Table 1). TPM was injected intraperitoneally (i.p.) at doses ranging from 2.5–60 mg/kg 30 min before hypoxia‐induced seizures in postnatal day 10 (P10) old rat pups (at P10‐P12 the rodent brain corresponds to the brain development of a human term neonate). TPM reduced the seizures in a dose‐dependent fashion with a calculated median effective dose (ED_50_) of 2.1 mg/kg. Maximum antiseizure effects were observed at 15 (75%) and 30 (80%) mg/kg, but the effect decreased at 60 mg/kg, indicating a truncated dose response. In a study in P15 rats, in which SE was induced by lithium–pilocarpine, TPM (10 or 50 mg/kg, i.p.) did not suppress behavioral seizures, did not shorten continuous polyspike activity in the EEG, and did not reduce total seizure time, cumulative seizure time, number of seizures, duration of SE, or spike frequency when compared with controls.^29^ However, chemically induced seizures (or SE) are not an etiologically valid model of neonatal seizures. We currently plan to evaluate TPM's antiseizure effects in a physiologically validated P11 rat model of asphyxia‐induced neonatal seizures, in which we previously reported antiseizure efficacy of PB and midazolam at clinically relevant doses.^30^

**TABLE 1:** Neuroprotective and adverse outcome‐modifying effects of TPM in neonatal animal models of hypoxia–ischemia (HI) or birth asphyxia. See text for chemical models of neonatal seizures.

Study	Model, species, and postnatal age	Dose of TPM and time of administration	Effect on neonatal seizures	Disease‐modifying effects				
				Period/age after initial insult examined	Neuroprotective effect	Effect on cognitive impairment	Effect on behavioral abnormalities	Effect on seizure susceptibility
Koh and Jensen^21^ and Koh et al.^22^	Hypoxia‐only, P10 rats	2.5–60 mg/kg before hypoxia or 30 mg/kg after hypoxia	Yes (when given before hypoxia)	20 days; 2^nd^ hit with kainate at P30	Reduction in kainate induced CA3 damage	NT	NT	Prevention of increased susceptibility to kainate
Noh et al.^23^	HI, P7 rats	(1) Either 20–100 mg/kg before and after HI or (2) two injections only after HI	NT	8 weeks	Yes	Yes	NT	NT
Mikati et al.^24^	Hypoxia‐only, P10 rats	30 mg/kg from P0‐P21	NT	P81	Yes (Ca1)	Yes	NT	NT
Schubert et al.^25^	HI, P2–P5 piglets	Loading dose 20 or 50 mg/kg (1 h after HI), maintenance dose 10 or 20 mg/kg/day over 2 days	No	68 h after treatment	Yes (frontal, temporoparietal, and occipital cortices, as well as striatum and hippocampus)	NT	Reduction of neurologic deficit	NT
Liu et al.^26^	HI, P7 rats	Loading dose 40 mg/kg (immediately after HI), then 10 mg/kg maintenance dose twice daily for 2 days	No	3, 21, and 22 days after HI	No (cortex, CA1, CA3, dentate gyrus, striatum, subcortical white matter)	NT	No (foot‐fault test)	NT
Liu et al.^27^	HI, P7 rat	BT 29°C over 3 h, starting 3 h after HI; TPM (30 mg/kg) given before delayed TH	NT	P15 and P35	Yes (only TH + TPM): reduced damage in cortex, striatum, and hippocampus)	NT	Yes (forepaw response to vibrissae stimulation)	NT
Landucci et al.^28^	HI, P7 rats	10 or 20 mg/kg immediately and 2 h after hypoxia	NT	P10	Yes (reduction of infarct size at 20 mg/kg)	NT	NT	NT
Landucci et al.^28^	HI, P7 rats	10 mg/kg immediately and 2 h after hypoxia, administered together with TH	NT	P10	Yes (reduction of infarct size larger than with TPM without TH)	NT	NT	NT

Abbreviations: HI, hypoxia‐ischemia; NT, not tested; P, postnatal day; TH, therapeutic hypothermia.

### 2.3 Neuroprotective and adverse outcome‐modifying effects of TPM in neonatal animal models

The various studies that examined the effects of TPM on the adverse outcome of HI and neonatal seizures in animal models are summarized in Table 1. In addition to studying the effect of TPM on hypoxia‐induced neonatal seizures in P10 rat pups, Koh and Jensen^21^ assessed the subsequent susceptibility of rats to kainate‐induced seizures 4 days (P14) and 20 days (P30) after they were exposed to hypoxia, as well as CA3 neuronal injury at P30. In contrast to vehicle controls with hypoxia, in animals that had seizures suppressed by TPM during acute hypoxia, there were no long‐term increases in susceptibility to kainate‐induced seizures and seizure‐induced neuronal injury. In a subsequent study, Koh et al.^22^ reported that TPM (30 mg/kg) given for 48 h *after* hypoxia‐induced seizures prevents the increase in susceptibility to kainate‐induced hippocampal neuronal injury at P28/30, indicating that this neuroprotective effect occurs independent of TPM's anticonvulsant action. In a P7 rat model of HI, TPM administered over 48 h post‐injury significantly reduced brain lesion severity at 30 mg/kg but not 10 or 50 mg/kg, again indicating a truncated dose response at high doses.^31^ In apparent contrast, Noh et al.^23^ reported that administration of TPM (20–100 mg/kg) reduced the brain damage and subsequent cognitive impairment induced by transient HI (induced by unilateral carotid occlusion and transient hypoxia) in P7 rats at all examined doses. These effects were also observed when TPM was administered within 2 h after the insult. It should be noted that one P7 HI rat study^26^ found no neuroprotective effect of TPM when using a relatively high (40 mg/kg) loading dose (Table 1).

TPM administration (30 mg/kg daily) in rat pups (from P0–P21), which were exposed to hypoxia at P10, prevented hypoxia‐induced long‐term (P81) memory impairment (Morris water maze) as well as aggressivity (handling test).^24^ The hypoxia group receiving TPM also showed a trend toward reduced CA1 hippocampal cell loss. When newborn piglets were subjected to HI by transient occlusion of carotid arteries and hypotension, administration of TPM (loading dose 50 mg/kg; maintenance dose 20 mg/kg/day over 2 days) after HI led to a significant reduction of neuronal cell loss in the frontal, temporoparietal, and occipital cortices, as well as striatum and hippocampus.^25^

Hypoxia‐induced seizures in P10 rats were shown to induce a rapid increase in AMPA receptor signaling, which may play a critical role in epileptogenesis, that is, the later development of epilepsy in childhood after neonatal hypoxia.^32^ Injection of TPM (30 mg/kg, i.p.) immediately after hypoxia‐induced seizures attenuated the AMPA receptor potentiation and, as shown previously by the same group, prevented the subsequent increase in in vivo seizure susceptibility, indicating an antepileptogenic effect.^22^, ^32^

Liu et al.^27^ evaluated whether treatment with TPM increases the efficacy of delayed post‐HI hypothermia in a neonatal rat stroke model. P7 rat pups underwent right carotid artery ligation followed by 1.5 h of exposure to 8% oxygen. Fifteen minutes post‐HI, animals received injections of TPM (30 mg/kg) or vehicle. Cooling was initiated 3 h later (“delayed hypothermia”) in all animals for 3 h (in a 27°C incubator). Functional outcome (forepaw response to vibrissae stimulation) and pathology (morphometric lesion measurements) were evaluated at P15 and P35 (Table 1). Neither TPM nor delayed hypothermia alone conferred protection in this protocol. Combined treatment with TPM and delayed hypothermia improved both performance and pathological outcomes in P15 and P35 rats. This was the first preclinical study that showed that TPM improves the neuroprotective effect of TH.

Landucci et al.^28^ compared the neuroprotective effect of TPM with vs without TH in a P7 rat model of HI. Administration of TPM (20 mg/kg, i.p.) both immediately and 2 h after hypoxia significantly reduced the extent of the resulting cortico‐striatal lesion, whereas 10 mg/kg was ineffective. When TPM was administered together with TH (32°C for 4 h beginning 1 h after hypoxia), a marked neuroprotective effect was obtained with 10 mg/kg, which exceeded the effect obtained with TH alone. This finding substantiates the report of Liu et al.^27^ that TPM augments the neuroprotective effect of TH after HI insults.

The neuroprotective effect of TPM in neonatal rodents has also been reported for chemical convulsants. Zhao et al.^33^ induced a series of seizures by flurothyl in P10–P14 rat pups, followed by prolonged treatment with TPM (40 mg/kg once to twice daily). TPM treatment reduced the amount of seizure‐induced aberrant mossy fiber sprouting in the supragranular region. Furthermore, rats treated with TPM performed significantly better in the water maze test of spatial learning and memory than rats treated with saline. Sfaello et al.,^34^ who induced excitotoxic brain damage in neonatal (P5) mice and rats using the AMPA and kainate receptor agonist S‐bromowillardiine, reported that a subsequent injection of a single dose of TPM (1, 10, or 30 mg/kg, i.p.) induced dose‐dependent neuroprotection of both the cortical plate and underlying white matter lesions at P10. In contrast, diazepam, phenytoin (PHT), and carbamazepine (CBZ) were not neuroprotective in this model.

Different lines of evidence suggest that the neuroprotective effect of TPM is mediated by its antagonistic activity at glutamate receptors.^23^, ^31^ The latter mechanism may also be involved in the antiepileptogenic effects of TPM, which was reported by Suchomelova et al.^29^ in P15 rats, in that TPM (10 mg/kg) administered 20 or 40 min after pilocarpine completely prevented the development of spontaneous recurrent seizures, indicating a potent antiepileptogenic efficacy of TPM. It is important to note that TPM at a higher dose (50 mg/kg) did not prevent development of epilepsy at any time point tested, again indicating a truncated dose response (see also next section).

### 2.4 Is TPM neurotoxic in the developing brain?

Bittigau et al.^35^, ^36^ reported that treatment of neonatal rats with ASMs (PB, PHT, diazepam, clonazepam, vigabatrin, and valproate) causes excess apoptotic neurodegeneration in the developing rat brain. The authors suggested that these preclinical findings may present one possible mechanism to explain cognitive impairment and reduced brain mass associated with prenatal or postnatal exposure of humans to treatment with certain ASMs, which raised significant concerns for the use of such drugs in the treatment of neonatal seizures.^8^ In a subsequent study, Glier et al.^37^ reported that treatment of neonatal rats with anticonvulsant doses of TPM (5–20 mg/kg) does not induce apoptotic neurodegeneration. In line with the latter report, treating rat pups with TPM (30 mg/kg/day) from P0–P21 had no long‐term deleterious effects on memory, hyperactivity, or CA1 cell counts when compared with normal controls.^24^ Similar data were reported by Cha et al.^38^ Neurotoxic effects of TPM were observed only at doses of 50 mg/kg and above, that is, doses that were significantly higher than reported anticonvulsant ED_50_ doses in rodent seizure models.^37^ Thus, in contrast to several other ASMs, including PB, TPM has a favorable therapeutic index in terms of the separation between anticonvulsant activity and neurotoxic/proapoptotic effects in the developing rat brain. Furthermore, as discussed in Section 2.3, TPM is one of the few ASMs that exerts neuroprotective effects at anticonvulsant doses.

### 2.5 Pharmacokinetics of TPM in rodent models

The half‐life of TPM in adult rodents (2.5 h in rats) is considerably shorter than its half‐life in adult humans.^19^ In P7–P10 rat pups, the half‐life of TPM (~4.7 h) is considerably longer than in adult rats^39^ but still much shorter than in human neonates (see Section 3.3). Clark et al.^40^ determined the plasma levels of TPM in P7–P10 rat pups associated with neuroprotective activity in an HI model. Mean steady‐state maximum and average concentrations following 30 mg/kg TPM, i.p., were 31.3 and 16.8 μg/mL in Long Evans and 39.9 and 24.4 μg/mL in Sprague–Dawley pups. The average steady‐state plasma levels of TPM are in (or slightly above) the therapeutic plasma concentration range (5–20 μg/mL) determined in adult epilepsy patients at daily oral doses of 200–400 mg.^41^

For terminating seizures in an emergency setting, it is important how rapidly an ASM penetrates into the brain after parenteral administration. TPM has been reported to rapidly enter the rodent brain after i.p. administration.^16^ Similarly, following i.v. administration of 50 mg/kg TPM in a brain microdialysis study in rats, effective extracellular TPM concentrations were achieved within 15 min.^42^ At this time point, TPM levels were already above the TPM concentration range (0.65–1.13 μg/mL) determined by brain microdialysis during epilepsy surgery in patients after oral administration of clinically approved doses of this ASM.^43^ This indicates that effective TPM brain levels are achieved within a few minutes after i.v. administration.^44^

## 3 CLINICAL DATA

TPM is a broad‐spectrum ASM approved as an oral formulation for preventing or reducing the frequency of epileptic seizures (as monotherapy or adjunctive therapy), and for the prophylaxis of migraine.^45^, ^46^ For epilepsy, TPM is approved as an oral monotherapy for individuals 2 years of age or older with primary generalized onset tonic–clonic or focal‐onset seizures. In addition, it is sanctioned for adjunctive therapy in adults and pediatric populations from ages 2 to 16 years with primary generalized onset tonic–clonic seizures or focal‐onset seizures, and for those 2 years or older with seizures associated with Lennox–Gastaut syndrome. TPM has numerous off‐label applications, including the treatment of refractory SE^44^ and neonatal seizures.

### 3.1 Antiseizure effects of TPM in neonates

Silverstein and Ferriero^12^ reported survey results suggesting that TPM is a common off‐label add‐on agent for PB refractory neonatal seizures and is considered beneficial by the majority of pediatric neurologists in NICUs. Glass et al.^47^ were the first to report antiseizure effects of TPM in a retrospective cohort study in six newborns with neonatal seizures caused by ischemic and/or hemorrhagic brain injury that were refractory to standard treatment with PB or PHT (Table 2). TPM was administered as a suspension of crushed tablets via a nasogastric tube. The dose used was 10 mg/kg in five subjects and 3 mg/kg in one subject. No seizures were detected on follow‐up EEG in three subjects (including the newborn with 3 mg/kg TPM), and the frequency of seizures was reduced in a fourth newborn.

**TABLE 2:** Clinical studies on the antiseizure effect of topiramate in neonates with acute symptomatic seizures.

Study	Patient population	Number of patients	Study type	EEG confirmation of seizures	Dose of topiramate	Therapeutic hypothermia?	Outcome
Glass et al.^47^	Neonates with acute symptomatic seizures refractory to first‐line treatment (phenobarbital or phenytoin)	6	Retrospective cohort study	Yes (in 5/6)	10 mg/kg (except one patient with 3 mg/kg) by nasogastric tube	Not mentioned	In four of six neonates, apparent reduction (1) or no further seizures (3) occurred
Kundak et al.^48^	Neonates with acute symptomatic seizures (4, + 2 genetic) refractory to ASM treatment	6	Retrospective case study	Not described	6 mg/kg/day orally	No	Control of seizures in 2/6 neonates
Riesgo et al.^49^	Preterm neonates with acute seizures of unclear etiology refractory to ASM treatment (phenobarbital)	3	Case study	Yes	3.5–8 mg/kg/day orally	Not mentioned	Complete control of seizures in all neonates
Vawter‐Lee et al.^50^	Neonates with seizures of all etiologies refractory to first‐ and second‐line ASM treatment	75	Retrospective cohort study	Yes	Loading and maintenance dose 1–10 mg/kg/day orally	Yes (but no details given)	Efficacious in 46/75 (61%); 72% continued on TPM at hospital discharge
Zheng et al.^51^	Neonates with acute symptomatic seizures	7	Retrospective case study	Yes	2 mg/kg/day to 18 mg/kg/day	Not mentioned	Effective in 5/7 neonates
Sadeghvand et al.^52^	Neonates with seizures (75% idiopathic, 20% HIE) refractory to first‐line treatment (phenobarbital)	60^a^	Double‐blind cross‐sectional study	Yes (but no details given)	3–8 mg/kg/day orally	Not mentioned	No difference between topiramate, levetiracetam or additional phenobarbital in controlling the seizures (but no details regarding outcome measure)

Abbreviations: ASM, antiseizure medication; EEG, electroencephalogram; HIE, hypoxic‐ischemic encephalopathy.

^a^ After phenobarbital failed, neonates (20 per group) were randomized to (1) additional phenobarbital (5 mg/kg/day), (2) topiramate (3–8 mg/kg/day), and (3) levetiracetam (10–40 mg/kg/day).

In a subsequent retrospective case study by Kundak et al.,^48^ TPM (6 mg/kg/day orally) was administered for refractory seizures as an add‐on treatment for uncontrolled seizures in six patients, four of whom were asphyxiated and not treated with hypothermia (Table 2). TPM controlled the seizure activity in only two of these neonates, but the authors suggested that 10 mg/kg or higher doses of TPM may prove more effective.

Riesgo et al.^49^ reported the clinical off‐label use of TPM in three cases of refractory neonatal seizures of unknown etiology in preterm newborns (Table 2). In all cases, the seizures were completely controlled by adding TPM (3.5–8 mg/kg orally).

The largest retrospective cohort study on TPM was performed by Vawter‐Lee et al.,^50^ primarily aimed at evaluating safety, so limited efficacy data were provided. In 75 neonates with acute symptomatic seizures refractory to first‐ and second‐line ASM treatment, TPM, at loading and maintenance doses of 1–10 mg/kg orally, was felt to be efficacious in 61% of the neonates but no details were given (Table 2). Almost half of neonates received no additional ASMs after TPM initiation, and 72% of surviving neonates were continued on TPM at discharge.

Zheng et al.^51^ reported data from seven patients who received TPM for onset of seizures between the ages of 35 and 45 weeks of gestation (Table 2). In the initial response, defined at 24 h after administration, there was a decrease in clinical seizures confirmed with EEG in five of seven neonates.

Sadeghvand et al.^52^ recently reported the outcome of a double‐blind cross‐sectional clinical trial comparing the antiseizure efficacy of PB, TPM, and LEV (Table 2). Sixty neonates with seizures were treated i.v. with 10–40 mg/kg PB and, after this treatment failed to control the seizures, randomized to three groups with (1) oral PB maintenance (5 mg/kg/day), (2) oral TPM (3–8 mg/kg/day), or (3) LEV (10–40 mg/kg/day). The authors described that all three treatments were equally effective in controlling seizures but no details were provided regarding the outcome measure.

In most of these studies, TPM doses below 10 mg/kg were given and plasma drug levels were not determined. Thus, it is not clear whether drug plasma levels in the effective concentration range of TPM (5–20 μg/mL) were reached. Based on TPM plasma levels determined in 52 neonates with HIE treated with TH, in whom TPM was administered by nasogastric tube at 5 mg/kg on Day 1 and 3 mg/kg on Days 2–5 (which resulted in subtherapeutic TPM plasma levels in the majority of cases), a modified dosage schedule was designed to obtain more than 90% of patients with TPM concentrations within the therapeutic range after the first dose.^53^ The modified dosage schedule obtained consisted of a loading dose of 15 mg/kg on Day 1 and maintenance doses of 5 mg/kg for subsequent days. These calculations should be considered when planning future clinical trials with TPM in neonates.

### 3.2 Effects of TPM on the consequences of HIE and neonatal seizures

The promising neuroprotective and disease‐modifying effects of TPM reported in animal models of HI and neonatal seizures (Section 2.3) prompted two randomized controlled multicenter trials, in which TPM was added to TH and compared with TH alone in neonates with moderate to severe HIE.^54^, ^55^ In the pilot study of Filippi et al.,^54^ 44 term neonates with clinical and EEG signs of HIE were treated with TH plus TPM (10 mg/kg once a day for the first 3 days) or TH alone to evaluate the safety and efficacy of TPM. Whole‐body TH was started within 6 h after birth and continued for 72 h. TPM was administered by nasogastric tube at the beginning of hypothermia. Average plasma levels of TPM were about 6.5–7 μg/mL after the first administration and gradually increased to a mean value of around 12–13 μg/mL after the third dose administration. Notably, TPM levels were lower in neonates treated with PB than those who did not receive PB. The number of neonates who needed treatment for acute symptomatic seizures with PB was not different between TH (*n* = 14) and TH + TPM (n = 14). Compared to TH alone, TPM did not reduce the combined frequency of mortality and severe neurological disability (determined at 18–24 months of age). Furthermore, based on structural brain magnetic resonance imaging (MRI), the predominant pattern of injury and the incidence of moderate–severe brain injury were similar in both groups. However, consistent with the antiepileptogenic effect of TPM described in animal models (Section 3.2), there was a lower prevalence of epilepsy in newborns co‐treated with TPM, since epilepsy occurred in 3 of 21 (14.3%) of the treated group (at 18–24 months) vs 7 of 23 (30.4%) of the control group (*p* = .211). Filippi et al.^54^ considered this trend worthy of further studies, considering that this finding was obtained in a pilot study and that TH alone does not reduce the incidence of epilepsy developing after HIE.^56^

Nuñez‐Ramiro et al.^55^ conducted a similar but larger randomized placebo‐controlled double‐blinded multicenter trial with the primary objective to determine if TPM prophylaxis would reduce seizure activity and improve neurologic outcome, in addition to TH. Neonates (*n* = 106) were randomly assigned to TPM (54) or placebo (52) at the initiation of TH (on average 2.5–5 h after birth). TPM was administered via nasogastric tube at a bolus dose of 5 mg/kg and a maintenance dose of 3 mg/kg/day for 5 days. The TPM group had a lower incidence of seizures in the first 24 h of hypothermia (TPM, 14/54 [25.9%] vs placebo, 22/52 [42%]), needed less additional ASMs, and had lower mortality (TPM, 5/54 [9.2%] vs placebo, 10/52 [19.2%]); however, these results did not achieve statistical significance. TPM plasma level determinations showed that TPM achieved a therapeutic range of 5–20 mg/L in only 37.5% and 75.5% of the patients at 24 and 48 h, respectively, that is, TPM levels were subtherapeutic in the first 24 h when seizure activity is typically maximal in HIE.^57^ A significant association between serum TPM levels and seizure activity (*p* = .016) was observed. The authors concluded that the results warrant further studies with higher loading and maintenance dosing of TPM.

### 3.3 Pharmacokinetics of TPM in neonates

Filippi et al.^58^ determined the pharmacokinetics (PK) of TPM following nasogastric administration in nine neonates with HIE and how the PK were affected by TH and PB (Table 3).

**TABLE 3:** Pharmacokinetic parameters of topiramate in neonates and adults. The pharmacokinetics of oral topiramate were determined with immediate‐release preparations (Topamax).

	Dose	C_max_ (μg/mL)	T_max_ (h)	T_0.5_ (h)	CL/F (mL/kg/h in neonates or mL/min in adults)	V_d_ (L/kg)	References
Neonates with HIE							
With TH (*n* = 52)	Bolus 5 mg/kg, maintenance 3 mg/kg/d for 5 days (nasogastric)	4.2	9.8	54.1	19.7	–	Nuñez‐Ramiro et al.^55^
With TH; all (*n* = 9)	5 mg/kg once a day for 3 days (nasogastric)	17.9	3.8	35.6	15.4	–	Filippi et al.^58^
With deep TH (*n* = 3)	5 mg/kg once a day for 3 days (nasogastric)	17.9	4.0	48.8	15.7	–	Filippi et al.^58^
With mild TH (*n* = 6)	5 mg/kg once a day for 3 days (nasogastric)	18.7	4.1	29.0	13.9	–	Filippi et al.^58^
With PB (*n* = 4)	5 mg/kg once a day for 3 days (nasogastric)	15.4	3.1	29.5	17.9	–	Filippi et al.^58^
Without PB (*n* = 5)	5 mg/kg once a day for 3 days (nasogastric)	19.9	4.4	42.9	13.4	–	Filippi et al.^58^
Adults							
Healthy volunteers (*n* = 28)	100–1200 mg single dose orally	1.7–28.7	1.8–4.3	18.7–23.0	22.5–36.1	–	Doose et al.^59^
Healthy volunteers (*n* = 35)	Two doses of 100 mg orally 12 h apart	4.6	1.4	37.1	ND	–	Lambrecht et al.^60^
Healthy volunteers (*n* = 12)	100 mg single dose orally	1.8	1.35	41.18	20.3	–	Clark et al.^13^
Healthy volunteers (*n* = 12)	100 mg single dose i.v. (in Captisol®)	1.99	‐	42.3	22.2	1.06	Clark et al.^13^
Epilepsy patients, TPM monotherapy (*n* = 3)	400 mg/kg orally twice daily	5.5	1.7	ND	33.2	–	Sachdeo et al.^61^
Epilepsy patients, TPM plus CBZ (*n* = 12)	400 mg/kg orally twice daily	3.4	1.0	~15 h	63.7 (mL/min)	–	Sachdeo et al.^61^

Abbreviations: CBZ, carbamazepine; C_max_, maximum plasma concentration; CL/F, clearance; HIE, hypoxic–ischemic encephalopathy; ND, not determined; PB, phenobarbital; T_0‐5_, elimination half‐life; TH, therapeutic hypothermia; T_max_, time of maximum plasma concentration; TPM, topiramate; V_d_, volume of distribution.

Available data on TPM PK in normothermic neonates are limited to newborns of mothers treated with TPM and CBZ, in whom TPM half‐life was ~24 h.^62^ A similar half‐life of 29.5 h was determined by Filippi et al.^58^ for TPM in neonates with co‐administration of PB, whereas the half‐life was higher (42.9 h) in neonates without PB (Table 2), which may indicate increased metabolism of TPM by co‐administration with PB. This is also indicated by lower TPM clearance in neonates without PB. Similarly, it was shown previously that maternal intake of CBZ during pregnancy can induce fetal drug metabolism.^63^ Filippi et al.^58^ hypothesized that TH would increase TPM plasma levels, which is supported by the finding that the TPM half‐life was longer in neonates with deep TH (rectal or esophageal temperature of 30–33°C for 72 h) vs mild TH (33–34°C) (Table 3). However, in a large group of neonates treated with mild TH (rectal temperature of 33.5°C for 72 h), a TPM half‐life of 54.1 h was reported by Nuñez‐Ramiro et al.,^55^ which is similar to the half‐life of 48.8 h determined in neonates with deep TH by Filippi et al.^58^

At first glance, these half‐lives in neonates appear considerably longer than the half‐lives of TPM (19–23 h) determined in healthy adult volunteers by Doose et al.^59^ However, later PK studies in adult humans reported half‐lives ranging from 37–42 h (Table 3), which is not different from the TPM half‐lives in neonates. It is important to note that all oral PK studies in adults shown in Table 3 were performed with an immediate release preparation of TPM, whereas longer effective half‐lives were reported for an extended‐release formulation.^60^ As in neonates, co‐administration with enzyme‐inducing ASMs such as CBZ or PHT decreases the half‐life of TPM in adults (Table 3).

One study in adult volunteers used an i.v. formulation of TPM.^13^ This allowed determination of the volume of distribution of TPM, resulting in a value of 1.06 L/kg (Table 3), which indicates that TPM is distributed to all tissues and retains higher tissue than plasma levels. Indeed, rapid and extensive brain distribution of TPM has been shown both in animals and patients with epilepsy.^44^

TPM is not highly bound to plasma proteins (9%–17%) and not extensively metabolized, but is mainly eliminated through the kidneys with about 70%–80% of the administrated dose eliminated unchanged in the urine.^64^ Thus, although drug metabolism is lower in neonates than adults,^65^ this will not have a major impact on TPM elimination.

### 3.4 Safety of TPM in neonates

In all clinical studies of TPM in neonates reviewed in Sections 3.1, 3.3, TPM was reported to be safe, not associated with any serious adverse effects. In one of the largest neonatal studies, metabolic acidosis, which is a known but rare adverse effect in adult patients, resulting from carbonic anhydrase inhibition,^66^ was observed in only 2 of 75 neonates and nephrolithiasis in 1 of 75 neonates.^50^ In two other large neonatal studies with TPM, no significant alteration in blood pH was reported.^54^, ^55^ Indeed, no adverse effects were directly attributable to TPM and, consequently, no neonates were discontinued because of TPM adverse effects in either trial. TPM did not cause significant acidosis requiring treatment, despite presumably significant systemic illness (cardiac, renal, hepatic) as would be expected from neonates with moderate to severe HIE (about one‐half received cardiopulmonary resuscitation and more than one‐third received epinephrine).

However, a single‐institution case series suggested an association between TPM administration and subsequent development of necrotizing enterocolitis (NEC) in 4 of 10 preterm and late preterm neonates whose refractory seizures were treated with TPM.^67^ This prompted a multicenter retrospective cohort study that included 75 neonates who received TPM to treat seizures.^50^ The study demonstrated that the development of NEC after treatment with TPM was rare (4%) and within the baseline rate of NEC in severely ill neonates, thus refuting the report by Courchia et al.^67^

Treatment of neonates or infants with PB has been reported to adversely affect later‐life cognition,^68^ which could be a consequence of its neurotoxic effects on the developing brain (see Section 2.4). It is thus important to note that the treatment of neonates with TPM (plus TH) did not adversely affect cognition (determined at 18–24 months of age) in comparison to TH alone.^54^

Because most neonates with seizures have acute symptomatic seizures due to disorders (such as HIE, stroke, intracranial hemorrhage) that resolve within days, ASMs are not routinely continued beyond a few days or after NICU discharge.^69^ For the small minority of infants with neonatal‐onset epilepsy, (e.g., neonates with brain malformations or other disorders with risk of recurrent seizures), clinicians may continue TPM into infancy and beyond. TPM is used routinely for older infants, is well tolerated, and the adverse effects are well characterized. In a systematic review of 23 studies, TPM was rarely discontinued for adverse effects and there were low rates of severe adverse effects.^70^ A long‐term open‐label study of enteral TPM (25–60 mg/kg/d) as adjunctive therapy in 284 infants <2 years old provided additional data regarding safety of TPM in infants.^71^ They found that a low CO_2_ occurred in 40% with persistent metabolic acidosis in 23%, anorexia occurred in ~30% after weeks to months of treatment, and nephrolithiasis was observed in 6% of patients but was only symptomatic in one patient (0.3%). Notably, the latter study included infants receiving one or more ASMs in addition to TPM, so there may have been a contribution of other ASMs to these adverse effects.

## 4 NEW INTRAVENOUS FORMULATIONS OF TPM

At present, an injectable formulation of TPM is not clinically approved for human use. Nasogastric administration of TPM suspensions (as used for clinical treatment of neonatal seizures and SE in adults) is associated with slow onset of action and variable bioavailability.^15^, ^44^ TPM is only poorly water soluble and not stable in aqueous solutions.^15^ Therefore, different excipients have been used to increase the solubility and stability of TPM.

### 4.1 TPM dissolved by Captisol®

James Cloyd and coworkers (University of Minnesota) have developed a solubilized TPM formulation using a cyclodextrin matrix, Captisol.^13^ Captisol, a proprietary brand owned by Ligand Pharmaceuticals (San Diego, CA) is a patent‐protected, polyanionic beta‐cyclodextrin derivative (Figure 2), the chemical structure of which was rationally designed to enable the creation of new products by significantly improving solubility, stability, bioavailability, and dosing of active pharmaceutical ingredients.^72^ Cyclodextrins such as Captisol form drug inclusion complexes that are water‐soluble^73^; following systemic administration, the drug is released from the inclusion complex. In 2013, the company (CURx) developing the Captisol‐based injectable formulation of TPM received an ODD from the FDA for treatment of partial onset or primary generalized tonic–clonic seizures for hospitalized epilepsy patients or epilepsy patients being treated in an emergency care setting who are unable to take oral TPM. Initially, the TPM formulation was developed with the long‐term goal of evaluating its safety and efficacy in newborns with neonatal seizures,^13^ but—based on FDA regulations—parenteral products containing Captisol are not indicated for use in patients under 2 years of age because of insufficient toxicological knowledge in the pediatric population.^74^ As pointed out by Cloyd et al.,^75^ there are several FDA‐approved injection products containing Captisol that are prescribed for children as young as 2 years of age, that is, Vfend I.V. (voriconazole), Noxafil (posaconazole), and Veklury (remdesivir). In fact, Veklury is used in infants as young as 28 days of age (weighing at least 3 kg). However, to our knowledge, there are no data on the efficacy and safety of Captisol‐containing drug formulations in severely ill neonates shortly after birth. As pointed out recently^74^ the nephrotoxic potential of Captisol–although generally considered low—may be higher in critically ill neonates with compromised kidney function.

The PK and safety of the Captisol‐based TPM formulation have been examined in adult volunteers^13^, ^76^, ^77^ and adult patients with epilepsy and migraine.^14^ In these studies, the injected TPM solution contained 10 mg TPM per milliliter of a 10% solution of Captisol; details on osmolarity and pH of this solution were not provided. No studies on this formulation in the pediatric population are available in the public domain. Currently, Ligand Pharmaceuticals develops this TPM formulation as a parenteral replacement therapy for adult patients with epilepsy who cannot swallow the oral formulation.

### 4.2 TPM dissolved by meglumine

In contrast to Captisol, there are no safety issues or age restrictions with the excipient meglumine (Figure 2), a highly tolerable FDA‐approved amino sugar.^15^, ^44^, ^74^ Rundfeldt et al.^15^ were the first to use meglumine to prepare an aqueous solution of TPM. As indicated in Figure 2, the polyol (numerous hydroxyl groups) structure of meglumine and that of TPM strongly favors hydrogen bond interactions. Thus, the various oxygen atoms of the fructopyranose ring of TPM as well as the oxygens in the SO_2_ group could interact with the hydrogens from the numerous hydroxy groups in meglumine in hydrogen bonding, thus forming a meglumine–TPM complex that is highly water soluble.^15^ Such complex or supramolecular adduct formation with meglumine has been shown previously for several water‐insoluble drugs, such as, for instance, some nonsteroidal anti‐inflammatory drugs.^78^

We have demonstrated that by adding low amounts of meglumine to water, TPM is rapidly dissolved up to high drug concentrations.^15^ Indeed, meglumine proved to be much more effective for dissolving TPM than Captisol. Not only does meglumine markedly increase the water solubility of TPM, but it also increases the stability of the solution at room temperature. PrevEp (Bethesda, MD) is currently developing the meglumine‐based parenteral formulation of TPM for the treatment of neonatal seizures and received ODDs for this indication in both the United States and Europe. This formulation was shown to be safe and effective during first‐in‐human i.v. administration in an adult patient with epilepsy.^79^ In the latter study, a solution of 10 mg TPM per milliliter in a 1% aqueous solution of meglumine was used. For use in neonates, a solution of 20 mg TPM per milliliter in a 2% aqueous solution of meglumine will be used to reduce the injection volume. The osmolality of this solution is 161 mOsm/kg of solvent, which is below the normal osmolality range in plasma (275–295 mOsm/kg) but can be easily enhanced by adding NaCl or glucose. Based on the alkaline properties of meglumine,^80^ the pH of the 2% TPM solution is 9.5, which is acceptable for i.v. infusion of relatively small volumes.^81^, ^82^ A randomized, controlled, double‐blind multicenter clinical trial of TPM for neonatal seizures is currently being planned by the authors and many collaborators.

## 5 CONCLUSIONS AND FUTURE PERSPECTIVES

Based on the preclinical and pilot clinical data, TPM is currently among the most attractive ASMs for the treatment of neonatal seizures in newborns refractory to PB, because of multiple mechanisms of action and potential neuroprotective effects. However, its current off‐label use is restricted by the lack of an FDA‐approved parenteral formulation of TPM. An added advantage of TPM compared to most other ASMs is its neuroprotective and disease‐modifying effect and the lack of neurotoxicity at therapeutic doses. Future prospective clinical trials should be designed to test TPM efficacy in neonatal seizures refractory to PB as well as analyze efficacy compared with other more frequently used ASMs, such as PB, PHT, or midazolam. Furthermore, the potentially beneficial effects of TPM on the adverse outcome of PA/HIE with associated seizures should be examined in such trials. For such studies, clinical availability of an i.v. formulation of TPM with an excipient that is suitable for use in neonates will be vital.

## AUTHOR CONTRIBUTIONS

Wolfgang Löscher and Janet S. Soul contributed equally to refining the research questions, executing methodological procedures, and writing of the manuscript.

## FUNDING INFORMATION

No funding was received for this review.

## CONFLICT OF INTEREST STATEMENT

W. Löscher is cofounder and CSO of PrevEp, Inc. (Bethesda, MD, USA). He has received in the past 5 years consultancy fees from Lundbeck, Angelini, Clexio, Selene, Axonis, SynapCell, Sintetica, ND Capital, Atlas Venture, Cogent Biosolutions, Ovid, Idorsia, and Addex. J. S. Soul receives annual royalties from UpToDate.

## ETHICS STATEMENT

We confirm that we have read the Journal's position on issues involved in ethical publication and affirm that this report is consistent with those guidelines.

## Data Availability

No primary data were collected for this study. The original data extraction is available from the corresponding author upon reasonable request.
